# Ginsenoside compound K induces apoptosis in nasopharyngeal carcinoma cells *via* activation of apoptosis-inducing factor

**DOI:** 10.1186/1749-8546-9-11

**Published:** 2014-04-02

**Authors:** Carmen Ka-Man Law, Hoi-Hin Kwok, Po-Ying Poon, Chi-Chiu Lau, Zhi-Hong Jiang, William Chi-Shing Tai, Wendy Wen-Luan Hsiao, Nai-Ki Mak, Patrick Ying-Kit Yue, Ricky Ngok-Shun Wong

**Affiliations:** 1 Department of Biology, Faculty of Science, Hong Kong Baptist University, Hong Kong SAR, China; 2 Faculty of Chinese Medicine, Macau University of Science and Technology, Macau SAR, China; 3 Center for Cancer and Inflammation Research, School of Chinese Medicine, Hong Kong Baptist University, Kowloon, Hong Kong SAR, China

## Abstract

**Background:**

Nasopharyngeal carcinoma (NPC) has a high incidence rate in Southern China. Although there are conventional therapies, the side effects and toxicities are not always tolerable for patients. Recently, the tumoricidal effect of ginsenosides on different cancer cells has been studied. This study aims to investigate the anti-cancer effect of ginsenosides on NPC cells and their underlying mechanism.

**Methods:**

The cytotoxicity of ginsenosides on NPC cell line HK-1 was measured by MTT assay. Apoptosis was detected by propidium iodide staining followed by flow cytometry. A xenograft tumor model was established by injecting nude mice with HK-1 cells. The activation of caspases and apoptosis-inducing factor (AIF) were evaluated by Western blot analysis. Nuclear translocation of AIF was also studied by immunofluorescence staining. Mitochondrial membrane potential was measured by JC-1 dye using flow cytometry.

**Results:**

Four ginsenosides, 20 (S)-Rh_2_, compound K (CK), panaxadiol (PD) and protopanaxadiol (PPD), induced apoptotic cell death in HK-1 cells in a concentration-dependent manner. CK inhibited HK-1 xenograft tumor growth most extensively and depleted mitochondrial membrane potential depolarization and induced translocation of AIF from cytoplasm to nucleus in HK-1 cells. In addition, depletion of AIF by siRNA abolished CK-induced HK-1 cell death.

**Conclusion:**

Ginsenoside CK-induced apoptosis of HK-1 cells was mediated by the mitochondrial pathway and could significantly inhibit tumor growth *in vivo*.

## Background

Nasopharyngeal carcinoma (NPC) is a head and neck cancer with a distinctive ethnic and geographic distribution. In Southern China, NPC has a high incidence of about 25–30 per 100,000 persons per year, in contrast to the low incidence of less than 1 per 100,000 persons per year was recorded in Western countries [1]. The common treatment for NPC is radiotherapy. Besides the undesirable side effects of radiotherapy, the location of the tumor also leads to complications after treatment. Chemotherapy is an alternative in treating NPC but resistance to conventional drugs is a challenge. Therefore, new multiple regimens such as radiochemotherapy and combination treatments with adjuvant drugs are being studied [2].

Ginsenosides are a group of saponin glycosides, which contribute to the pharmacological effects of ginseng [3]. More than 40 ginsenosides have been separated and identified from ginseng [4,5] and can be classified into three groups: protopanaxadiols (PPD) (*e.g*., Rb_2_, Rc, Rd, Rg_3_, and Rh_2_), protopanaxatriols (PPT) (*e.g*., Re, Rf, Rg_1_, Rg_2_, and Rh_1_), and oleanolic acid derivatives [6,7]. Structure-activity relationship studies on different ginsenosides and their anti-cancer effects have been demonstrated that ginsenosides with a sugar moiety at C-6 (PPT-type) exhibit less cytotoxicity than those without a sugar moiety at C-6 (PPD-type) [8]. In the last few years, ginsenosides were reported to be responsible for the vasorelaxation, antioxidation, anti-inflammation, anti-angiogenesis and anti-cancer effects of ginseng [9,10]. Ginsenosides PPD and Rh_2_ exhibited anti-proliferative effects on intestinal and glioma cell models [11-13]. Apoptosis induction by different ginsenosides was also demonstrated on human astrocytoma cells, human epidermal carcinoma cells, HeLa cells, and HT-29 colon cells [14-18]. Compound K (CK) is the major metabolite of PPD-type ginsenosides, and is transformed by intestinal bacteria [19]. CK is rapidly absorbed in the gastrointestinal tract and is retained for a long time in rat plasma [20,21]. The anti-angiogenic effect of CK was also reported [14,16,22]. Although the anti-cancer effects of ginsenosides have been studied extensively in other cancer models, the effect of ginsenosides on NPC is unknown. Several natural compounds extracted from plants could induce apoptosis in NPC through the mitochondria-dependent pathway. For example, capsaicin (EC_50_ ~ 300 μM) from hot chili peppers [23], aloe emodin (EC_50_ 100 μM) [24], and rhein (EC_50_ 180 μM) [25] isolated from the rhizome of rhubarb, induced depletion of mitochondrial membrane potential and subsequent AIF release in NPC-derived cell lines. However, ginsenosides and especially CK are more potent (EC_50_ 15 μM) than these natural compounds. Although caspase-dependent apoptosis induced by CK was reported in other cancer cell-lines [26-28], cell type-specific intracellular signaling might account for the discrepancy observed. The adjuvant effect of ginsenosides has been demonstrated by increasing chemotherapy efficacy [15] and patient survival rates [29,30].

This study aims to investigate the anti-cancer effects and action mechanism of ginsenosides on NPC cells.

## Methods

### Ginsenosides

High-performance liquid chromatography-purified ginsenosides as standard compounds (purity >98%) were purchased from Fleton Natural Products (Chengdu, China). Stock solutions of PPD (20 mM), CK (40 mM), and 20 (S)-Rh_2_ (40 mM) were prepared in dimethyl sulfoxide (DMSO) (Sigma Aldrich, St. Louise, MO, USA), while PD (20 mM) was prepared in absolute ethanol.

### Cell culture and drug treatments

NPC cell line HK-1 was maintained in RPMI 1640 medium (Gibco, Grand Island , NY, USA) supplemented with 10% fetal bovine serum (FBS) (Gibco) and 1% penicillin and streptomycin (Gibco) at 37°C in a humidified incubator with 5% CO_2_. HK-1 cells were starved in medium with 1% FBS for 24 h before drug treatment. Cells were treated with indicated concentrations of ginsenosides for different times in medium supplemented with 1% FBS.

### Cell viability assay

Cell viability was determined by the 3-(4,5-dimethylthiazol-2-yl)-2,5-diphenyltetrazolium bromide (MTT) assay. Briefly, HK-1 cells (1 × 10^4^ cells/well) were seeded onto 96-well plates and incubated overnight. Cells were starved in medium with 1% FBS for 24 h and then subjected to different treatments for another 24 h. After that, MTT solution (USB, Cleveland, OH, USA) was added into each well to a final concentration of 0.5 mg/mL and incubated for 3 h. The culture medium was then removed and DMSO was added to solubilize the purple formazan product. Absorbances at wavelengths of 540 and 690 nm were measured by a microplate reader (ELx800, Biotek, Winooski, VT, USA).

### Cell cycle analysis

HK-1 cells (1.5 × 10^5^ cells/well) were seeded onto 6-well plates and incubated overnight. Cells were starved in medium with 1% FBS for 24 h and then treated with different ginsenosides for 24 h. Cells were harvested, washed with PBS (Gibco) twice, and fixed in 70% ethanol at −20°C. The cells were then stained with propidium iodide solution (Sigma-Aldrich) containing RNase A (1 mg/mL) (Roche, Mannheim, Germany). Cell-cycle analysis was performed with the FACSCalibur Flow Cytometer (BD Biosciences, San Jose, CA, USA) and the data were analyzed with the Cell Quest and the Modfit LT Version 3.0 software (Verity Software House, Topsham, Maine, USA).

### Western blot analysis

After drug treatment, cytosolic and nuclear lysates were extracted with the NE-PER Nuclear Protein Extraction Kit (Millipore, Bedford, MD, USA) according to the manufacturer’s protocol. The cytosolic fraction was extracted with cytoplasmic lysis buffer (1× cytoplasmic lysis buffer, 0.5 mM DTT, 1:1000 dilution of inhibitor cocktail) while the nuclear fraction was extracted with nuclear extraction buffer (1× nuclear extraction buffer, 1:1000 dilution of inhibitor cocktail). Protein concentrations were determined with the Bio-Rad Dc protein assay kit (Bio-Rad, Hercules, CA, USA). Equal amounts of protein samples were separated by SDS-PAGE and transferred onto a nitrocellulose membrane. The membrane was then probed with primary antibodies (anti-AIF) (Cell Signaling, Beverly, MA, USA) and subsequently incubated with secondary antibodies (HRP-conjugated goat anti-rabbit IgG) (Invitrogen). After washing with 0.1% TBS-T (USB), the membrane was visualized by an enhanced chemiluminescence detection system (Bio-Rad). For the cytosolic fraction, protein expression was compared with β-actin (Sigma-Aldrich). For the nuclear fraction, lamin A/C (Santa Cruz Biotechnology, Santa Cruz, CA, USA) was used for normalization.

### Xenografts in nude mice

Male BALB/c nude mice were purchased from the Animal Services Centre of Chinese University of Hong Kong. For the animal study, HK-1 cells were harvested and washed twice with PBS. For each site of injection, 3 × 10^6^ HK-1 cells were suspended in 100 μL serum-free RPMI-1640 culture medium and mixed with Matrigel in a 1:1 ratio (BD Biosciences). The cell-matrigel mixture was inoculated subcutaneously into the left and right flanks of 6–7 week-old nude mice. When the tumors were palpable (8 days after injection), the tumor-bearing animals were randomly divided into two groups (four mice per group). In group 1, mice were treated with 10 mg/kg/day CK orally (CK was mixed in a 0.5% carboxymethyl cellulose [CMC] suspension). In group 2, mice were treated with 0.5% CMC orally as the control. Tumor sizes were measured daily and calculated using the formula (L × W^2^)/2 mm^3^ (L = length; W = width). The experiment was performed according to the Animals (Control of Experiments) Ordinance (Hong Kong) and followed the Hong Kong Baptist University’s guidelines on animal experimentation. The tumor inhibition (%) was calculated as follows:

Tumor inhibition (%) = (Tumor volume of control group − Tumor volume of CK-treated group)/(Tumor volume of control group).

### Detection of mitochondrial membrane potential

HK-1 cells were incubated with 5 μg/mL JC-1 dye (Invitrogen) for 30 min. After that, cells were trypsinized and resuspended in PBS for flow cytometry analysis. JC-1 monomers and J-aggregates were detected by a flow cytometer on the FL1 and FL2 channels, respectively. The mitochondrial membrane potential is presented by the 580/530 nm ratio.

### Immunofluorescence assay

HK-1 cells were seeded on a glass coverslip at a density of 2 × 10^5^ cells/well in a 6-well plate and incubated overnight. Cells were starved with 1% FBS medium for 24 h and then treated with or without CK for another 8 and 24 h. The medium was then removed and the glass cover slips were washed with PBS. After that, cells were fixed with 4% paraformaldehyde (Sigma Aldrich) for 10 min followed by washing with PBS three times. Cells were permeabilized with 0.2% of Triton X-100 for 10 min followed by washing with PBS. Cells were probed with anti-AIF antibody in 3% BSA (Sigma Aldrich) overnight at 4°C and then secondary antibody (PE-conjugated goat-anti-rabbit IgG) (Invitrogen) for 2 h at room temperature. After washing with PBS, the coverslip was incubated with DAPI (0.5 g/mL) (Invitrogen) for 5 min. Coverslips were mounted with fluorescence mounting medium on slides and were subjected to examination and image capture by an Olympus FV1000 confocal scanning laser microscope (Essex, UK).

### Transfection of small interference RNA (siRNA)

HK-1 cells were seeded onto 6-well plates overnight, cells were then transfected with AIFM1 specific siRNA (50 nM) (Ambion, Austin, TX, USA) using Lipofectamine RNAiMAX transfection reagent (Invitrogen) in antibiotic-free RPMI-1640 culture medium. Drug treatment was performed 48 h after transfection.

### Statistical analysis

All data were presented as mean ± standard deviation (S.D). Comparisons were subjected to Student’s *t*-test or Kruskal-Wallis One Way Analysis of Variance (ANOVA) followed by Dunnets *post hoc* test for multiple comparisons. Statistical significance was accepted at *P* < 0.05.

## Results

### Ginsenosides 20(S)-Rh_2_, CK, PD, and PPD exhibited cytotoxicities towards HK-1 cells

**Figure 1:**
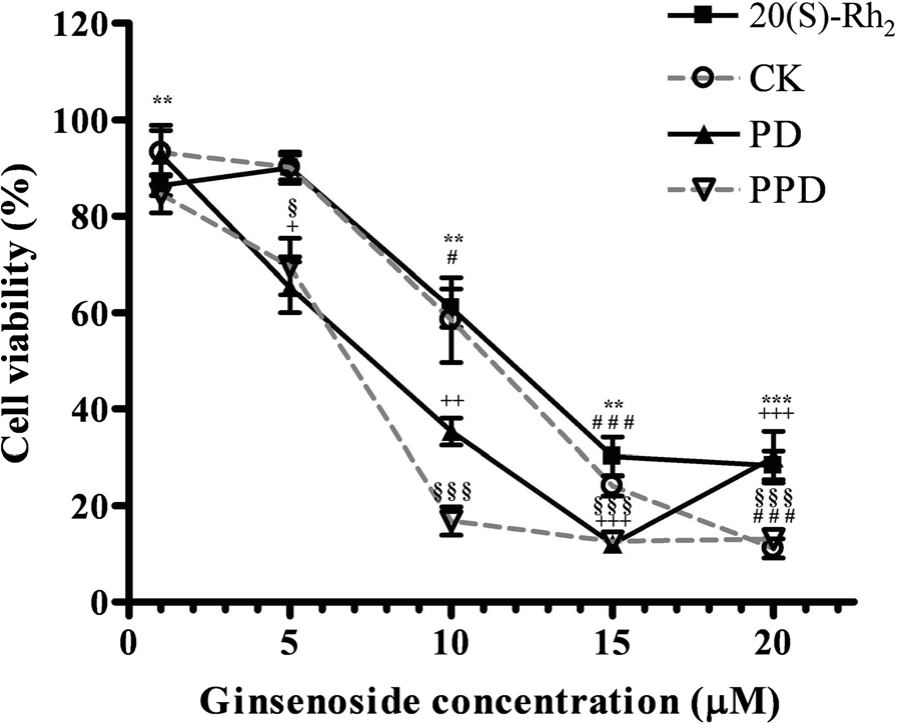
**Cytotoxic effect of 20 (S)-Rh**_**2**_**, CK, PD and PPD on HK**-**1 cells. **Cells were treated with different ginsenosides for 24 h in 1% FBS supplemented RPMI 1640 medium. The cell viability was determined by MTT assay and expressed as a percentage of solvent control in each experiment. Results were presented as mean ± SD from three independent experiments. **P < *0.01* and ***P < *0.001* 20 (S)-Rh_2_ compared to the DMSO control group. #*P* < *0.05* and ###*P* < *0.001* CK compared to the DMSO control group. +*P* < *0.05*, ++*P* < *0.01* and +++*P* < *0.001* PD compared to the ethanol control group. §*P* < *0.05* and §§§*P* < *0.001* PPD compared to the DMSO control group.

Using the MTT assay, ginsenoside 20(S)-Rh_2_, CK, PD, and PPD (1–20 μM) treatment inhibited growth of HK-1 cells in a dose-dependent manner (Figure 1). The IC_50_ of 20(S)-Rh_2_, CK, PD, and PPD, on HK-1 cells was 12, 11.5, 8, and 7 μM, respectively. Different concentrations of 20(S)-Rh_2_ (15 μM), CK (15 μM), PD (10 μM), and PPD (10 μM) were selected for subsequent studies. These data suggested that ginsenosides possess a cytotoxic effect on HK-1 cells.

### Ginsenosides induced apoptosis in HK-1 cells

**Figure 2:**
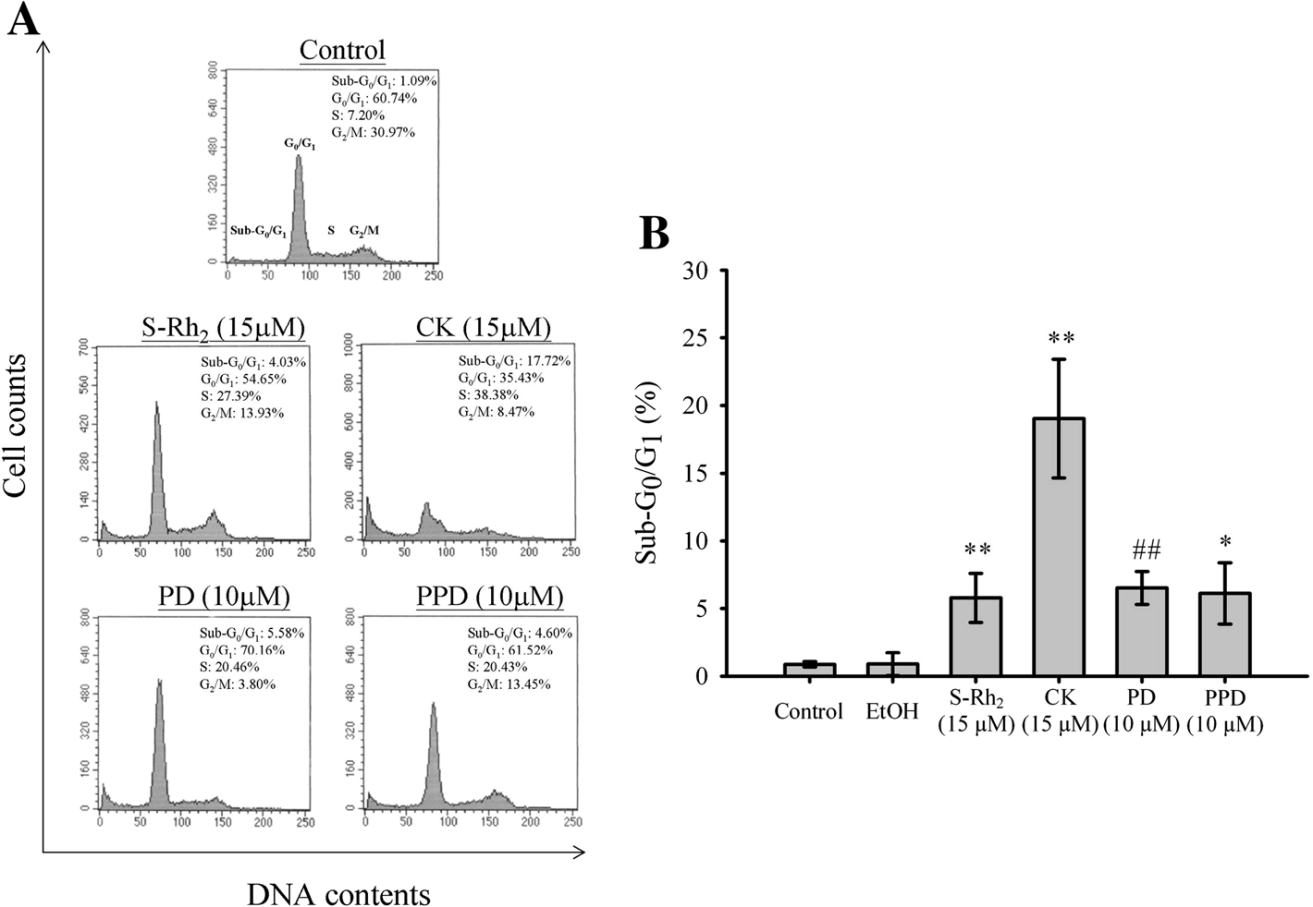
**Induction of apoptosis on ginsenosides**-**treated HK**-**1 cells. **HK-1 cells were treated with 20(S)-Rh_2_ (15 μM), CK (15 μM), PD (10 μM) and PPD (10 μM) for 24 h in 1% FBS RPMI 1640 medium. The cell cycle was then evaluated by propidium iodide staining and flow cytometric analysis. The data were analyzed by the Cell Quest and the Modfit LT Version 3.0 software to determine the percentage of cells in different cell cycle phases. Apoptosis was examined by comparing the sub-G1 population in HK-1 cells treated with or without ginsenosides. **(A)** Representative histograms of three independent experiments. **(B)** Statistical analysis on the percentage of sub-G_0_/G_1_ populations. Results were presented as mean ± SD from three independent experiments. *P < *0.05* and **P < *0.01* compared to the DMSO control group. #*P* < *0.05* compared to the ethanol control group.

The sub-G_1_ phase population represents cells in apoptosis. As shown in Figure 2A and 2B, ginsenosides 20(S)-Rh_2_, CK, PD, and PPD-treated HK-1 cells had a sub-G_1_ population of 4.0, 17.7, 5.6, and 4.6%, respectively. Ginsenosides can significantly induce apoptotic cell death in HK-1 cells.

### Ginsenosides induced caspase activation in HK-1 cells

**Figure 3:**
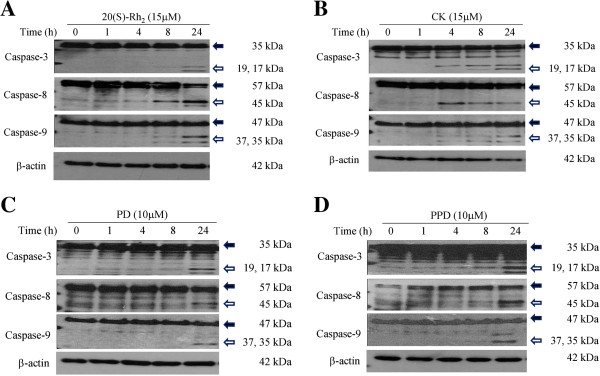
**Activation of caspase-3, -8 and -9 after ginsenosides treatment on HK-1 cells.** HK-1 cells were treated with ginsenosides** (A) **20(S)-Rh_2_ (15 μM), **(B)** CK (15 μM), **(C)** PD (10 μM) and **(D)** PPD (10 μM) in 1% FBS supplemented RPMI 1640 medium. Cells lysates were harvested at indicated time points for Western blot analysis using antibodies against both pro and active forms of caspase-3, -8 and -9. The expression of the pro and active forms was indicated by solid and hollow arrows, respectively. β-actin was used as a protein loading control.

Caspase-3, -8, and -9 were all activated by selected ginsenosides at different time points in HK-1 cells (Figure 3). In the case of 20(S)-Rh_2_ (Figure 3A) and CK (Figure 3B), treatment for 8 and 24 h activated the caspase-3, -8, and -9. In contrast, activation of the caspase cascade by PD (Figure 3C) and PPD (Figure 3D) occurred around 24 h after drug treatment. Moreover, earlier and stronger activation of caspase-8 was observed in 20(S)-Rh_2_- and CK-treated HK-1 cells when compared with PD- and PPD-treated cells. This implies that 20(S)-Rh_2_- and CK-induced apoptotic cell death in HK-1 cells may be mediated through the mitochondrial pathway.

### CK attenuated HK-1 xenograft tumors *in vivo* and induced caspase-independent apoptosis

**Figure 4:**
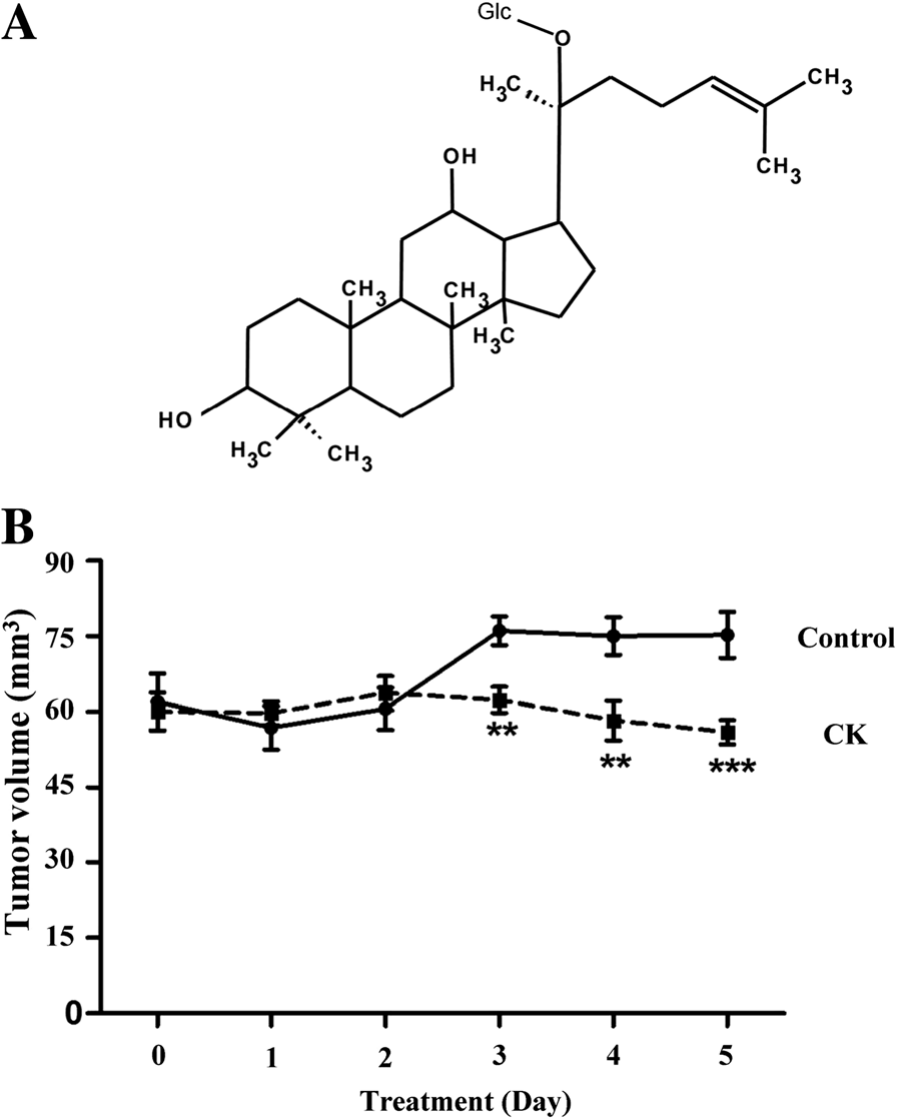
**CK attenuated the HK**-**1 tumor growth *in vivo*. (A)** Chemical structure of ginsenoside-CK. **(B)** The HK-1 xenograft tumor model was established by injecting 3 × 10^6^ cells subcutaneously to the left and right flanks of nude mice, respectively. Animals (4 mice per group) were treated with 10 mg/kg/day CK or 0.5% CMC (as control) orally for 5 days when tumors were palpable. Tumor sizes were measured daily. **P < 0.01 and ***P < 0.001 compared to the control group.

Among the four tested ginsenosides, we previously demonstrated the moderate cytotoxic effect of CK towards HK-1 cells. Additionally, CK induced a relatively high sub-G_1_ phase population and early activation of caspase cascade when compared with other ginsenosides. As CK is the most abundant metabolite of PPD-type ginsenosides, we selected ginsenoside CK (Figure 4A) as the representative ginsenoside in our further studies. In the animal experiment, tumor size in the CK-treated group was 25.6% lower than that in the control group at day 5. The average size of the eight tumors in the CK-treated group was 54.2 – 62.2 mm^3^*vs*. 70.6 – 79.8 mm^3^ in the control group (Figure 4B). No adverse effects were observed in either group of animals.

**Figure 5:**
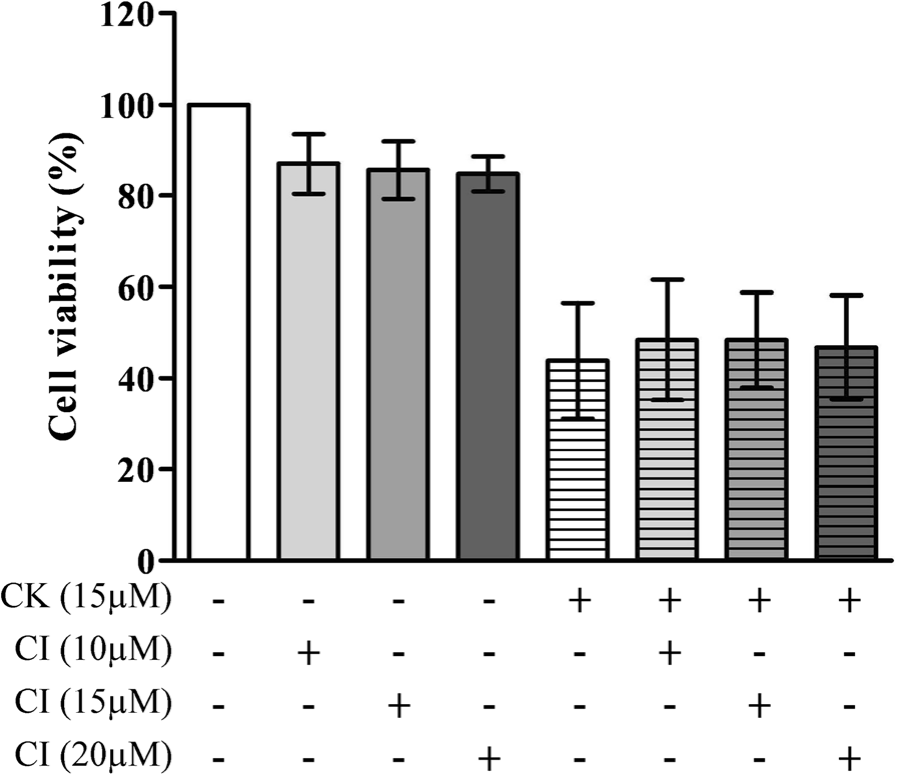
**Effect of caspase inhibitors on CK-induced HK-1 cell death.** HK-1 cells were pretreated with or without different concentrations of caspase cocktail inhibitor (CI) for 2 h followed by 24 h of CK (15 μM) treatment in 1% FBS supplemented RPMI 1640 medium. The cell viability was determined by MTT assay and expressed in percentage of solvent (DMSO) control. Results were presented as mean ± SD from three independent experiments.

In contrast to the western blot analysis on caspase activation, pretreatment with caspase inhibitors (Z-D (OMe) E (Ome) VD (OMe)-FMK, Z-IE (OMe) TD (OMe)-FMK, and Z-LE (OMe) HD (OMe)-FMK) together at 10, 15, or 20 μM did not reverse the cell death induced by CK (Figure 5). This indicates that the caspase activation was not the major pathway involved in the mechanism of CK-induced cell death. Thus, the caspase-independent apoptotic pathway was investigated.

### CK induced apoptosis-inducing factor (AIF) translocation and mitochondrial membrane depolarization

**Figure 6:**
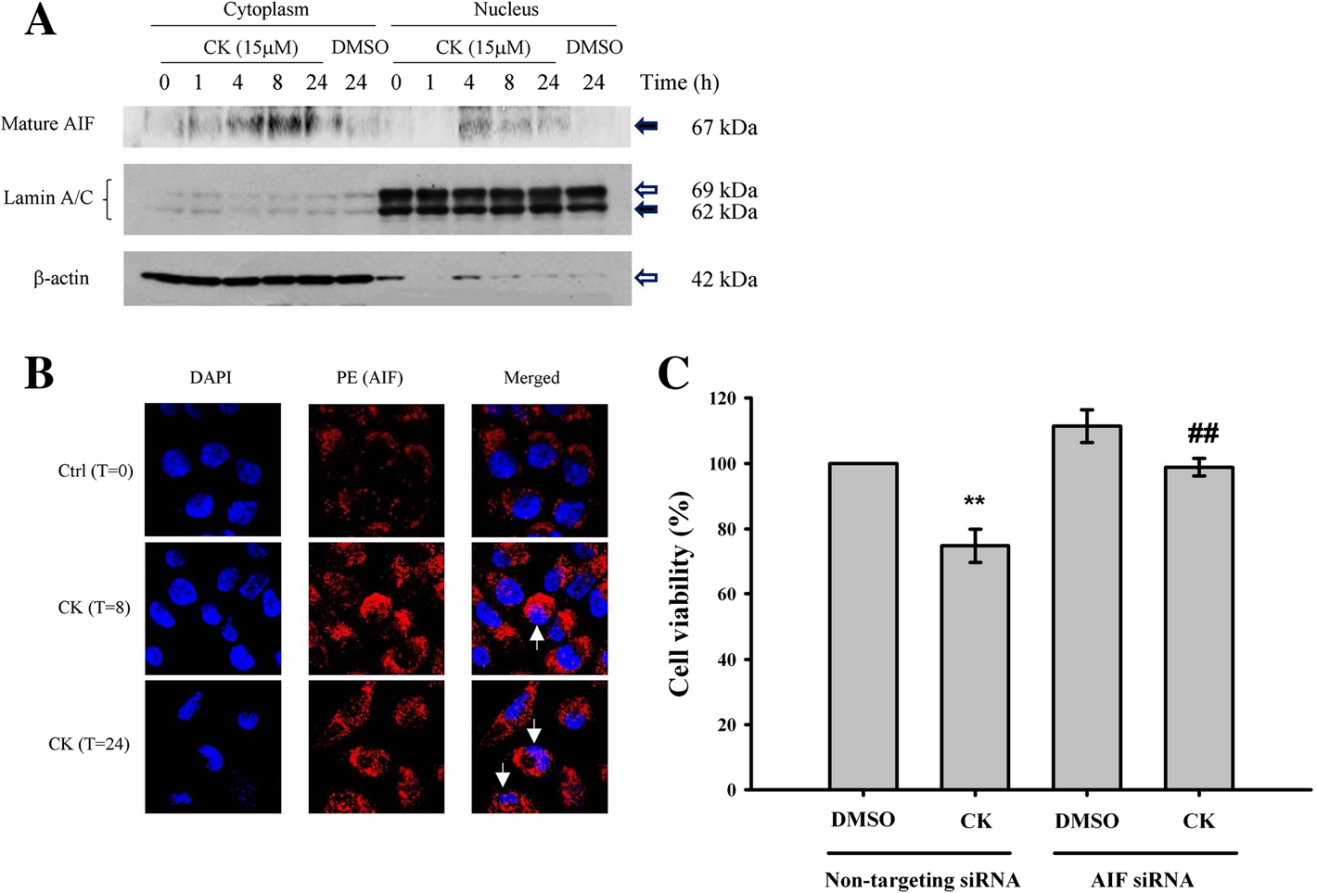
**CK**-**induced HK**-**1 cell death is dependent on AIF translocation from cytoplasm to nucleus.** Cells were starved in 1% FBS supplemented RPMI 1640 medium for 1 day followed by treatment with CK (15 μM) for the indicated time intervals. **(A)** Cell lysates were collected and fractionated with nuclear extraction kit into cytosolic and nuclear fractions. Western blot analysis was used to determine the protein expression of AIF, lamin A/C (nuclear marker) and β-actin (cytosolic maker). **(B)** Immunostaining of cells was performed to show the translocation of AIF from cytoplasm to nucleus. Cells were stained with anti-AIF antibody and a PE secondary antibody (red) to localize AIF. DAPI staining (blue) was used to show the nuclei. Images were captured by confocal laser scanning microscope. Arrows indicate cells with translocated AIF. **(C)** Cytotoxic effect of CK on HK-1 was reduced by AIF siRNA. After transfection of siRNA, HK-1 cells were treated with CK (1 μM) for 24 h in serum-free RPMI 1640 medium, cell viability was determined by MTT assay and expressed as a percentage of solvent control in each experiment. Results were presented as mean ± SD from three independent experiments. **P < *0.01* compared to the DMSO control group, ##*P* < *0.01* versus non-targeting siRNA CK group.

**Figure 7:**
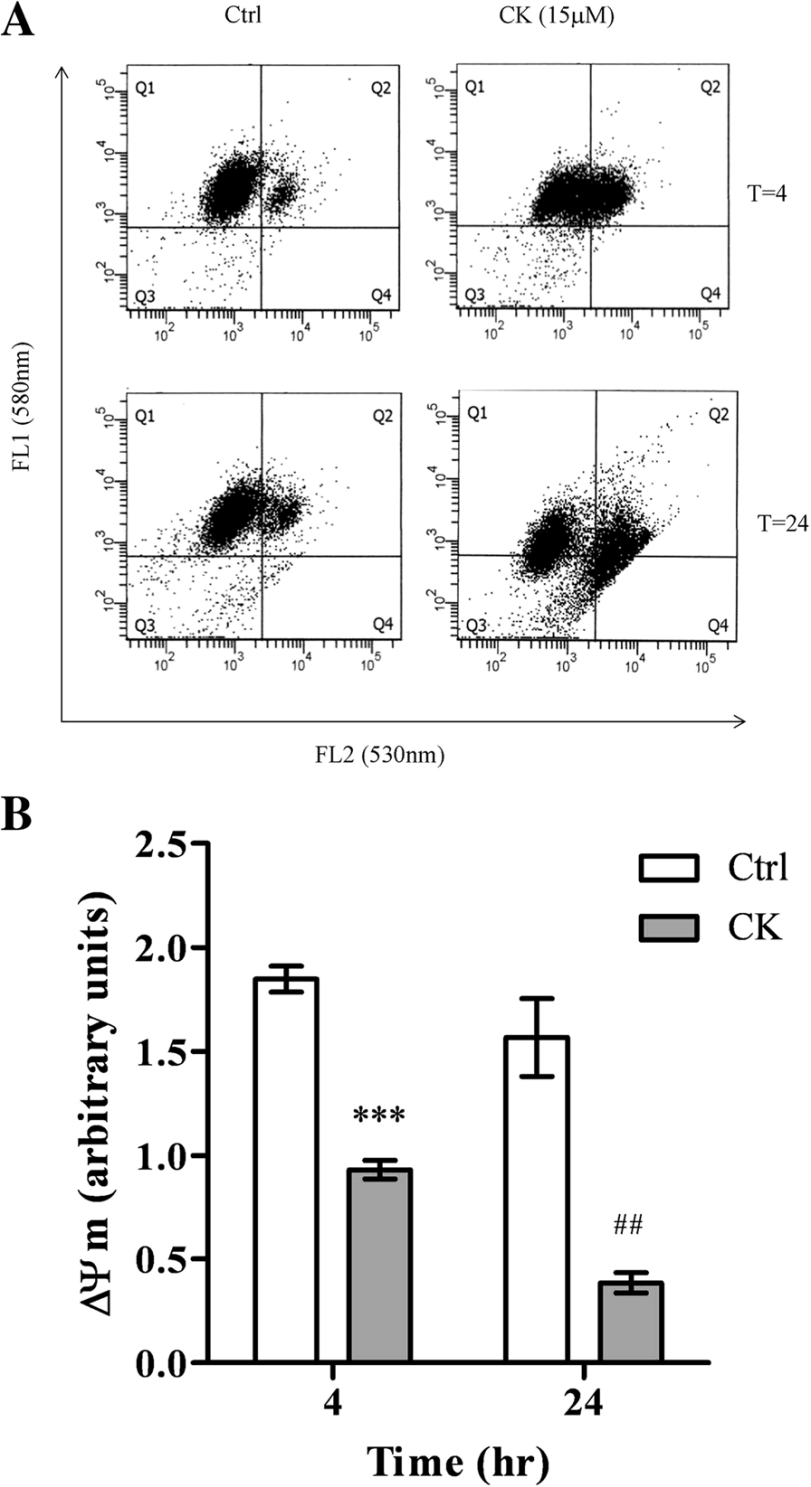
**Mitochondrial membrane potential of HK-1 cells after CK treatment. HK**-**1 cells were treated with CK (15 μM) for 4 and 24 h.** Mitochondrial membrane potential was examined by flow cytometry with JC-1 probe treatment. Aggregate form of JC-1 was present in mitochondria with high membrane potential, whereas its monomeric form appeared in mitochondria with low membrane potential. The aggregate and monomeric forms of JC-1 were detected by FL1 and FL2 detectors of flow cytometer, respectively. **(A)** Representative flow cytometry scatterplots were shown. The cell distribution in the Q4 area indicated the cells which had low mitochondrial membrane potential. **(B)** Mitochondrial membrane potential was presented in 580/530 nm ratio. The histogram showed the relative change of mitochondrial membrane potential to the control. ***P < *0.001* versus control at 4 h, ##*P* < *0.01* versus control at 24 h.

Translocation of AIF from mitochondria to nucleus is the key event of the caspase-independent apoptotic pathway [31]. Cells were treated with CK for 1, 4, 8, and 24 h. The mature form of AIF was significantly increased in both cytosolic and nuclear fractions after 4, 8, and 24 h treatments (Figure 6A). In addition, AIF translocation into nucleus was detected by immunofluorescence staining after 8 and 24 h treatment of CK (Figure 6B). We further confirmed that CK-induced apoptosis was dependent on the activation of AIF, siRNA of AIF was employed (Additional file 1: Figure S1). The cytotoxic effect of CK was significantly reduced by AIF siRNA (Figure 6C), which demonstrated the crucial role of AIF in CK-induced HK-1 cell death. Translocation of AIF requires the opening of mitochondrial pores. Therefore, mitochondrial membrane potential was determined by flow cytometry. Depolarization of the mitochondrial membrane potential was observed as early as 4 h after CK treatment with the potential decreased by nearly one-fold. Further depolarization was found after 24 h treatment (Figure 7A and 7B). This implied that CK-induced cell death in HK-1 cells was mitochondrially mediated.

## Discussion

Ginsenosides were reported to exhibit anti-proliferative, anti-metastatic, and anti-angiogenic activities in different *in vitro* and *in vivo* tumor models [10,32-35]. However, different ginsenosides induced diverse biological effects on different models due to structural differences. The number of sugar moieties were found to mediate ginsenosides activity by altering hydrophilicity. Moreover, aglycone ginsenosides (*i.e*., CK, PPD, and PPT) showed higher cytotoxicity than glycosides. This property of ginsenosides also mediated their affinity towards different molecular targets. CK is the major metabolite of all PPD-type ginsenosides in both rat and human plasma [20,21,31]. Aside from its tumoricidal effects, CK was shown to have neuroprotective, hypoglycemic, and antidepressant-like effects in mice, and enhancement of type I procollagen levels in ultraviolet-A-irradiated fibroblasts [36-39]. In the present study, HK-1 cells had a similar response towards 20(S)-Rh_2_, CK, PD, and PPD, and ginsenoside CK showed the most potent sub-G_1_ phase induction.

Apoptosis is a common type of cell death induced by anti-cancer drugs. Ginsenosides can induce apoptosis in different cancer models including human astrocytoma cells, HT-29 colon cells, A431 cells, and HeLa cells [14,17,18,40]. Apoptosis is mainly induced by a caspase cascade or translocation of AIF [41]. There are two pathways of caspase activation, which are the cell surface death receptor pathway (extrinsic) and mitochondria-initiated pathway (intrinsic). Caspase-3 is the “execute” caspase for the apoptotic induction, while caspase-8 and caspase-9 are the critical caspases and signify the activation of the extrinsic and intrinsic pathways, respectively [42]. In our study, we demonstrated apoptosis induction and caspase activation of ginsenosides in NPC cells. And pretreatment with caspase inhibitors did not reverse the cell death of CK-treated cells. This indicated that CK-induced cell death was caspase-independent. Besides inducing apoptosis, caspase activation was involved in other cellular responses, such as differentiation or cell migration [43]. Therefore, the CK-activated caspase cascade did not participate in the apoptotic execution.

**Figure 8:**
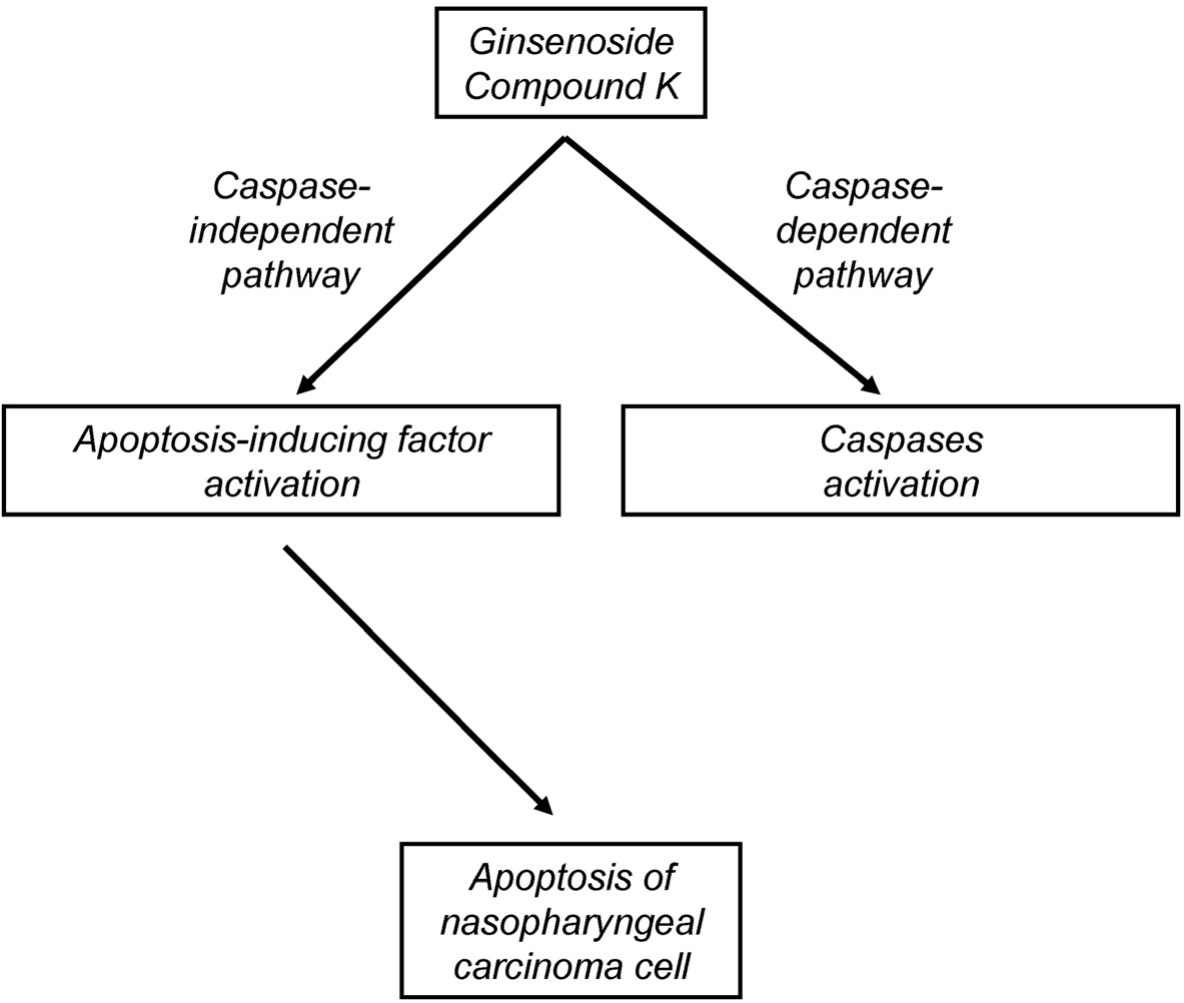
**Schematic diagram of ginsenoside CK**-**induced caspase**-**independent apoptosis on NPC cell.** Ginsenoside CK could activate both AIF translocation and cleavage of caspases, but the induction of apoptosis was only dependent on AIF activation.

Apart from the caspase-dependent apoptotic pathway, there is a caspase-independent apoptotic pathway in which AIF translocates from cytoplasm to nucleus [44,45]. AIF is a flavoprotein inducing DNA damage, chromatin condensation, and DNA degradation [31,46], which is normally present in mitochondria and will translocate into nucleus upon apoptotic induction. In the present study, translocation of AIF and depolarization of mitochondrial membrane potential were induced by CK treatment in HK-1 cells (Figure 8). This implied that CK induced apoptotic cell death of HK-1 cells *via* depolarization of mitochondrial membrane potential and activation of AIF.

## Conclusion

Ginsenoside CK-induced apoptosis of HK-1 cells was mediated by the mitochondrial pathway and could significantly inhibit tumor growth *in vivo*.

## Competing interests

The authors declare that they have no competing interests.

## Authors’ contributions

CCL, ZHJ, PYKY and RNSW designed and conceived the study. CKML, HHK, PYP, WCST, WWLH performed the experiments. CKML and HHK wrote the manuscript. All authors have read and approved the final manuscript.

## Supplementary Material

Additional file 1: Figure S1 Confirmation of knockdown of AIF. HK-1 cells were transfected with AIF-specific siRNA for 24 h. Expression of mature AIF was detected by Western blot analysis. β-actin was used as a protein loading control. Click here for file
