# Supplementary figures and images for: Ginsenoside compound K induces apoptosis in nasopharyngeal carcinoma cells via activation of apoptosis-inducing factor
Source: Chin Med. 2014 Apr 2;9:11. doi: 10.1186/1749-8546-9-11 (PMC4021625; doi:10.1186/1749-8546-9-11)

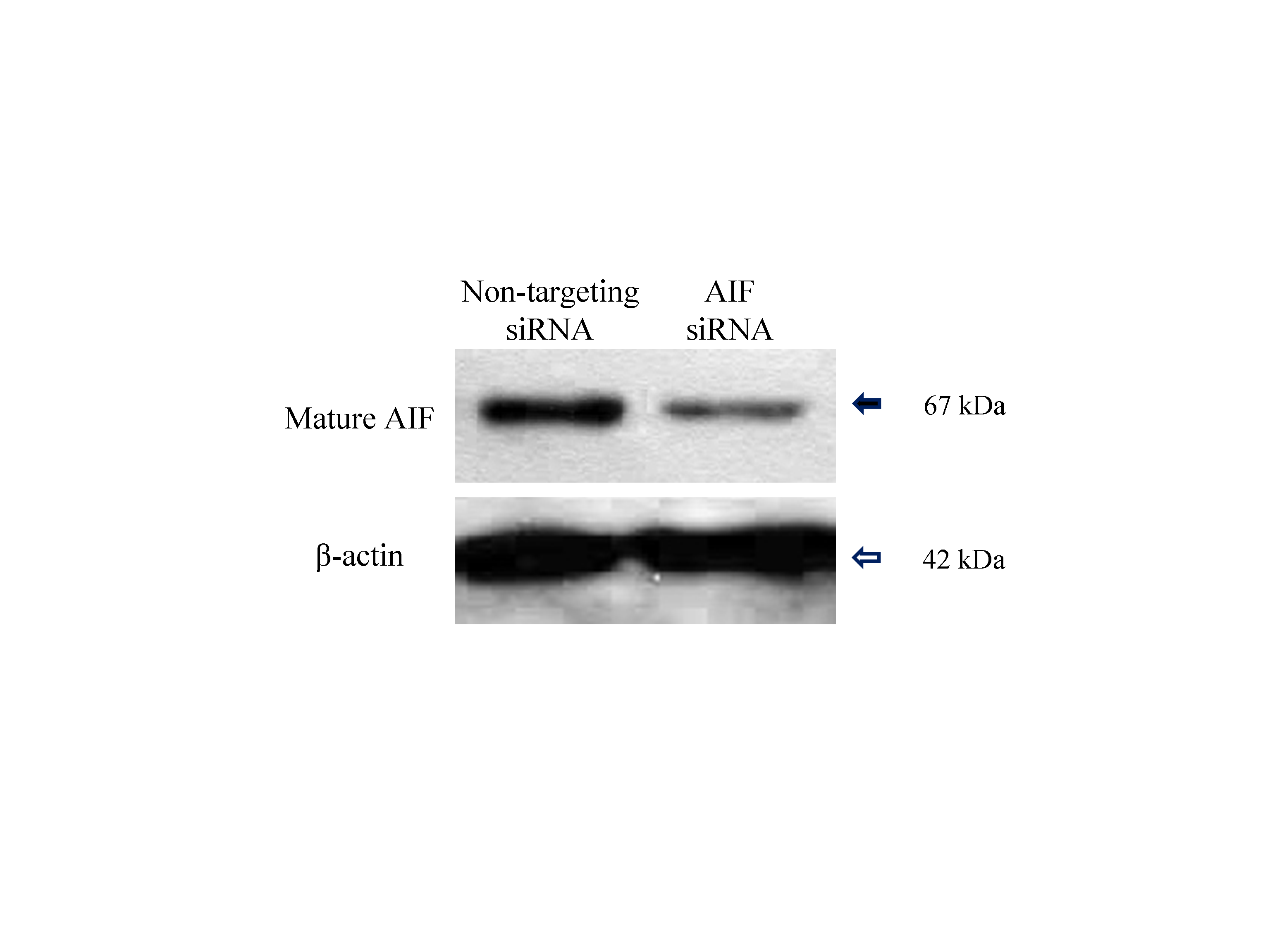

Supplement: Additional file 1: Figure S1 — Confirmation of knockdown of AIF. HK-1 cells were transfected with AIF-specific siRNA for 24 h. Expression of mature AIF was detected by Western blot analysis. β-actin was used as a protein loading control. [file 1749-8546-9-11-S1.tiff]
